# Differential effects of a post-anthesis fertilizer regimen on the wheat flour proteome determined by quantitative 2-DE

**DOI:** 10.1186/1477-5956-9-46

**Published:** 2011-08-04

**Authors:** Susan B Altenbach, Charlene K Tanaka, William J Hurkman, Linda C Whitehand, William H Vensel, Frances M Dupont

**Affiliations:** 1 United States Department of Agriculture, Agricultural Research Service, Western Regional Research Center, 800 Buchanan Street, Albany, CA 94710

**Keywords:** gliadins, glutenins, gluten proteins, nitrogen, sulfur

## Abstract

**Background:**

Mineral nutrition during wheat grain development has large effects on wheat flour protein content and composition, which in turn affect flour quality and immunogenic potential for a commodity of great economic value. However, it has been difficult to define the precise effects of mineral nutrition on protein composition because of the complexity of the wheat flour proteome. Recent improvements in the identification of flour proteins by tandem mass spectrometry (MS/MS) and the availability of a comprehensive proteome map of flour from the US wheat Butte 86 now make it possible to document changes in the proportions of individual flour proteins that result from the application of mineral nutrition.

**Results:**

Plants of *Triticum aestivum *'Butte 86' were grown with or without post-anthesis fertilization (PAF) and quantitative 2-dimensional gel electrophoresis (2-DE) was used to analyze protein composition of the resulting flour. Significant changes in the proportions of 54 unique proteins were observed as a result of the treatment. Most omega-gliadins, high molecular weight glutenin subunits (HMW-GS) and serpins as well as some alpha-gliadins increased in proportion with PAF. In contrast, alpha-amylase/protease inhibitors, farinins, purinins and puroindolines decreased in proportion. Decreases were also observed in several low molecular weight glutenin subunits (LMW-GS), globulins, defense proteins and enzymes. The ratio of HMW-GS to LMW-GS in the flour increased from 0.61 to 0.95 and the ratio of gliadins to glutenins increased from 1.02 to 1.30 with PAF. Because flour protein content doubled with PAF from 7 to 14%, most protein types actually increased in absolute amount (μg/mg flour protein). Data further suggest that flour proteins change with PAF according to their content of sulfur-containing amino acids Cys + Met.

**Conclusions:**

A 2-DE approach revealed changes in the wheat flour proteome due to PAF that are important for flour quality and immunogenic potential. The work forms a baseline for further studies of the effects of environmental variables on flour protein composition and provides clues about the regulation of specific flour protein genes. The study also is important for identifying targets for breeding programs and biotechnology efforts aimed at improving flour quality.

## Background

Each year wheat farmers make strategic decisions about the amount and timing of fertilizer applications that influence yield, grain quality, and economic returns for this major world crop. Such decisions may also impact local water and air quality. One well-documented effect of fertilization with nitrogen (N) is the increase in protein content. However, this depends in a complex way upon genotype, environment, timing and type of N application [1-7]. There is a strong inverse correlation between grain protein content and yield and early applications of nitrogen may increase plant growth and yield rather than grain protein [8-10]. In contrast, application of fertilizer at heading or anthesis under conditions of adequate soil moisture may be more effective in producing high yields of grain with high protein content [4,11]. High protein content is desirable for many wheat products and premiums are often paid for high protein bread wheat. However, mineral nutrition also affects protein composition, which impacts functionality, nutritional value, and immunogenic potential of the flour. In order to predict the effects of fertilizer applications on protein composition and identify breeding targets for improved flour quality, it is essential to understand the precise effects of fertilizer on the wheat flour proteome [12].

Wheat flour protein composition is complex [13]. The major flour protein types are identified by their tendency to partition into different solvent fractions [7,14]. Typically these are the water-soluble albumins, salt-soluble globulins, acid or alcohol-soluble gliadins, and a glutenin polymer that is partially soluble in acetic acid or alcohol. The proteins in each solubility fraction are encoded by multiple similar genes located at complex homeoallelic loci that are replicated in two genomes for durum wheat (A and B) and three genomes for bread wheat (A, B, and D). These proteins may differ in their functional and nutritional properties and in their ability to trigger allergies and the serious food intolerance celiac disease.

Fractionation studies showed that additional N was differentially partitioned into gliadins and glutenins, which increased in amount per mg of flour, compared to an albumin/globulin fraction that did not increase [15-19]. Protein fractionation followed by SDS-PAGE and RP-HPLC revealed more details of this response, detecting increased proportions of HMW-GS and omega-gliadins and decreased proportions of LMW-GS and albumins/globulins in response to added N [12,16,18]. Similar changes in protein proportions were observed when grain was produced under conditions of sulfur (S) deficiency [20-23]. Methods used in these analyses were not adequate to completely separate the flour proteins by type. Quantitative 2-DE also has been used to measure changes in individual flour proteins in response to mineral nutrition [15,16,20,24,25]. But results from most studies have been limited by the challenges involved in identifying individual proteins within the major gluten protein families by MS/MS [26,27].

A recent 2-DE MS/MS study of proteins in white flour from Butte 86, a US spring wheat cultivar, identified 157 distinct proteins in 233 spots that together comprised 93% of the flour protein [28]. The 157 proteins included five HMW-GS, 22 LMW-GS, 13 gamma-gliadins, seven omega-gliadins, 23 alpha-gliadins, three farinins, three purinins, three triticins, eight globulins, three grain-softness related proteins, 16 amylase/protease inhibitors, nine serpins, three tritins, one xylanase inhibitor, three beta-amylases, 33 enzymes and five other proteins. Improved proteomics techniques and the availability of numerous gene sequences from the cultivar Butte 86 made it possible to distinguish closely related proteins within the major protein groups and link them to gene sequences [28-31]. The study also demonstrated that multiple 2-DE spots, mainly in apparent charge trains, were likely to be the products of single genes. This finding made it possible to quantify the amount of each individual protein type in the flour [28]. In this paper, the improved proteomic methods were used to measure the effects of PAF on these 157 unique proteins.

## Results

### Analysis of flour produced with and without PAF

The effects of PAF on grain yield and flour properties were measured for grain harvested at maturity (Table 1). Bushel weight increased by 3.3%, but flour yield and moisture did not change. Flour protein content doubled from 7.0% to 14.0%. Standard flour quality measurements such as mixing time and mixing tolerance, did not change significantly, but loaf volume increased by 59%. PAF had little effect on flour carbon (C) content. However, N and S contents increased significantly and the ratio of N:S increased from 11 to 15. The S content of flour with and without PAF was >0.1%, indicating that S was not deficient in our experiment. As reported previously, the treatment with PAF had little effect on the duration of grain fill or grain size (data not shown) [1,15].

**Table 1:** Effect of PAF on grain yield, flour properties and CNS composition

	**- PAF**^**1**^	**+ PAF**^**1**^	**% Change**^**2**^
Bushel Weight (kg)	61.0 +/- 0.3	63.0 +/- 0.4	3.3*
Flour Yield (% dry weight)	67.5 +/- 1.2	68.8 +/- 0.6	1.9
Flour Moisture (%)	13.8 +/- 0.0	14.2 +/- 0.2	2.9
Flour Protein (%)	7.0 +/- 0.4	14.0 +/- 0.9	100.0*
Mix Time (min)	2.8 +/- 0.6	2.2 +/- 0.3	-21.4
Mix Tolerance^3,4^	2.3 +/- 0.6	3.7 +/- 0.6	60.9
Loaf Volume (ml)^3^	51.7 +/- 1.5	82.0 +/- 3.5	58.6*
% C	39.6 +/- 0.1	40.1 +/- 0.1	1.3
% N	1.27 +/- 0.06	2.50 +/- 0.15	96.9*
% S	0.12 +/- 0.00	0.17 +/- 0.01	41.7*
N:S	10.6 +/- 0.03	14.7 +/- 0.04	38.7*

^1 ^Mean and standard deviation from flour samples from three biological replicates.

^2 ^* indicates that change was significant, p < 0.02.

^3 ^Determined using 10 g flour samples.

^4 ^Recorded on a 1-4 scale with 4 having the greatest tolerance.

### 2-DE protein spots that change in volume with PAF

**Figure 1:**
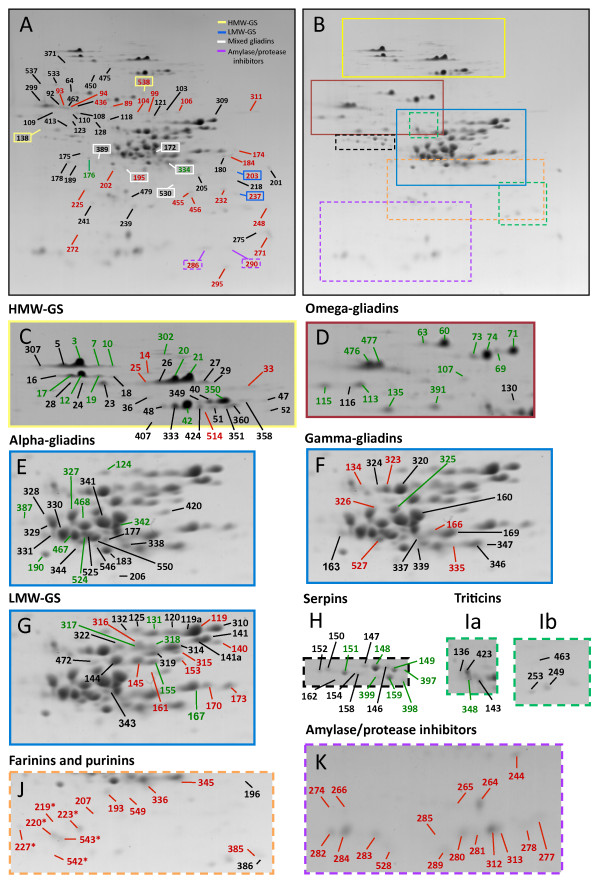
**Effect of post-anthesis fertilizer (PAF) on accumulation levels of individual wheat flour proteins**. Flour proteins from plants grown without (A) or with (B) PAF were analyzed by 2-DE. Boxes in B indicate protein classes that are depicted in panels C-K. The spots indicated in panel A fall outside the perimeters of the selected areas in panel B. The green and red numbers indicate spots that significantly increased or decreased, respectively, and the black numbers indicate spots that did not change with PAF. Spots containing the large subunits of triticin are indicated in panels Ia and the small subunits in Ib. Asterisks in panel J designate spots containing purinins. Protein sequences associated with spots that increased or decreased significantly are listed in Tables 2 and 3. The identities of the proteins that did not change significantly are listed in Additional file 1.

Total protein was extracted from white flour from grain produced with and without PAF and analyzed by quantitative 2-DE (Figure 1). Gel patterns among biological replicates and among treatments were highly reproducible. The average spot volume (SV) was determined for 373 spots from each treatment (Additional file 1). These accounted for 96.9% of total SV for the -PAF treatment and 97.7% of total SV for the +PAF treatment. Proteins in 231 spots were identified by tandem mass spectrometry (MS/MS) in the previous study of Dupont et al. [28]. Identified spots accounted for 89.7% of total SV for the -PAF treatment and 92.8% of total SV for the +PAF treatment. Of the 373 spots quantified by 2-DE, 155 spots had significant (p < 0.02) differences in SV under the two fertilizer regimens (Additional file 1). These spots accounted for approximately 50% of the total SV under both treatments. Fifty-one spots increased with PAF and 104 spots decreased with PAF (Additional file 1).

Among the spots that increased with PAF were 13 spots identified as omega-gliadins, 11 spots identified as HMW-GS, eight spots identified as alpha-gliadins, seven spots identified as serpins, five spots identified as LMW-GS, and one spot identified as gamma-gliadin (Figure 1, Table 2). Additional spots that increased were one that contained a mixture of alpha- and gamma-gliadins, one identified as triticin and one identified as malate dehydrogenase. Spots that increased with PAF were associated with 27 distinct protein sequences. Three other minor spots also increased with PAF but had volumes less than 0.1 and were not identified in Dupont et al. [28] (Additional file 1).

**Table 2:** 2-DE spots that increased significantly with PAF

Spot Number	**Protein Sequence**^**1**^
60	Omega-gliadin *Gli-B3 *TC11_288652^2^
63	Omega-gliadin *Gli-B3 *TC11_288652^2^
71	Omega-gliadin *Gli-B3 *TC11_288652^2^
74	Omega-gliadin *Gl-iB3 *TC11_288652^2^
69	Omega-gliadin *Gli-B3*^3^
73	Omega-gliadin *Gli-B3*^3^
107	Omega-gliadin Cys type TC_262770^2^
113	Omega-gliadin Cys type TC_262770^4^
115	Omega-gliadin Cys type TC_262770^4^
135	Omega-gliadin Gli-A3 Bu-D5^2^
391	Omega-gliadin Gli-A3 Bu-D5^5^
476	Omega-gliadin *Gli-D3*^3^
477	Omega-gliadin *Gli-D3 *[GenBank:AAT74547]^5^

12	HMW-GS Ax2* [GenBank:AAB02788]^2^
17	HMW-GS Ax2* [GenBank:AAB02788]^2^
19	HMW-GS Ax2* [GenBank:AAB02788]^2^
20	HMW-GS Bx7 [GenBank:CAA32115]^2^
21	HMW-GS Bx7 [GenBank:CAA32115]^2^
302	HMW-GS Bx7 [GenBank:CAA32115]^2^
350	HMW-GS 1By9 [GenBank:CAA43361]^2^
3	HMW-GS Dx5 [GenBank:ABG68042]^2^
7	HMW-GS Dx5 [GenBank:ABG68042]^2^
10	HMW-GS Dx5 [GenBank:ABG68042]^2^
42	HMWGS Dy10 [GenBank:P10387]^4^

342	Alpha-gliadin Bu-1^5,6^
468	Alpha-gliadin Bu-3^5,6^
467	Alpha-gliadin Bu-4^5,6^
327	Alpha-gliadin Bu-11^5^
387	Alpha-gliadin Bu-12^5^
524	Alpha-gliadin Bu-12^5^
190	Alpha-gliadin Bu-14^4^
124	Alpha-gliadin Bu-BQ807130^2,6^

148	Serpin Bu-1^2^
149	Serpin Bu-2^5^
397	Serpin Bu-2^2^
398	Serpin Bu-3^2^
399	Serpin Bu-3^2^
159	Serpin Bu-4^4,6^
151	Serpin Bu-5^2^

167	LMW-GS Bu-1^5^
131	LMW-GS Bu-3^2^
317	LMW-GS Bu-2/13^4^
318	LMW-GS Bu-2/13^5^
155	LMW-GS Bu-18^5^

325	Gamma-gliadin Bu-1or Bu-8^5,7^
334	Gliadin, mixed spot^8^
348	Triticin TC11_285558^5^
176	Malate dehydrogenase [GenBank:AAT64932]^2^

^1 ^Identifications are from Dupont et al. [28].

^2 ^MS/MS analysis in [28] indicated that spot contained only one protein.

^3 ^No peptides identified by MS/MS in [28]. Spot identified in [43].

^4 ^Predominant protein identified by MS/MS in [28]. Analysis indicated that spot also contained other proteins from the same class.

^5 ^Predominant protein identified by MS/MS in [28]. Analysis indicated that spot also contained proteins from a different class.

^6 ^Predominant protein sequence identified in only one spot.

^7 ^MS/MS data in [28] was not sufficient to discriminate among proteins.

^8 ^More than one protein was identified in spot in [28], but no one protein was predominant.

Among the spots that decreased significantly with PAF were 19 identified as alpha-amylase/protease inhibitors, 11 as LMW-GS, six as gamma-gliadins, six as farinins, six as purinins, five minor spots identified as globulins, five minor spots identified as HMW-GS, two spots identified as beta-amylase, two spots as chitinase and two as puroindoline (Figure 1, Table 3). Single spots identified as the large subunit of ADP glucose pyrophosphorylase, lipid transfer protein (LTP), thaumatin-like protein, triose phosphate isomerase, elongation factor EF1A, and glucose and ribitol dehydrogenase as well as one spot that contained a mixture of gliadins also decreased in SV with PAF. Spots that decreased with PAF were associated with 50 distinct protein sequences. Thirty-three other spots decreased significantly in SV, but were not identified by MS/MS in Dupont et al. [28]. All but one were minor spots with volumes less than 0.1 in the absence of PAF (Additional file 1).

**Table 3:** Individual 2-DE spots that decreased significantly with PAF

Spot Number	**Protein Sequence**^**1**^
289	Alpha-amylase inhibitor WMAI [PRF:223520]^2^
528	Alpha-amylase inhibitor WMAI [PRF:223520]^2^
283	Alpha-amylase inhibitor WDAI TC11_338524^3,4^
286	Alpha-amylase inhibitor WDAI [GenBank:AAV91972]^3,4^
312	Alpha-amylase inhibitor WDAI [SwissProt:P01085]^4,5^
313	Alpha-amylase inhibitor WTAI-CM1 TC11_340510^4,5^
280	Alpha-amylase inhibitor WTAI-CM2 [SwissProt:P16851]^3^
285	Alpha-amylase inhibitor WTAI-CM2 [SwissProt:P16851]^3^
264	Alpha-amylase inhibitor WTAI-CM3 [SwissProt:P17314]^3^
265	Alpha-amylase inhibitor WTAI-CM3 [SwissProt:P17314]^3^
266	Alpha-amylase inhibitor WTAI-CM16 [SwissProt:P16159]^2^
284	Alpha-amylase inhibitor WTAI-CM16 [SwissProt:P16159]^2^
274	Alpha-amylase inhibitor WTAI-CM17 [GenBank:CAA42453]^2^
282	Alpha-amylase inhibitor WTAI-CM17 [GenBank:CAA42453]^3^
244	Alpha-amylase/subtilisin inhibitor WASI [SwissProt:P16347]^2,4^
281	Protease inhibitor CMx1/CMx3 TC11_309398^3,4^
290	Protease inhibitor CMx1/CMx3 TC11_308146^3,4^
277	Chymotrypsin inhibitor WCI [GenBank:CAD19440]^2,4^
278	Chymotrypsin inhibitor WCI, mixed spot^6^

170	LMW-GS Bu-1^5^
119	LMW-GS Bu-3^2^
161	LMW-GS Bu-3^5^
237	LMW-GS Bu-3^2^
316	LMW-GS Bu-3^5^
140	LMW-GS Bu-4^2^
173	LMW-GS Bu-6^4,5^
145	LMW-GS Bu-7^3^
203	LMW-GS Bu-11^5^
315	LMW-GS Bu-11^3^
153	LMW-GS Bu-18^2^

326	Gamma-gliadin Bu-1^4,5^
166	Gamma-gliadin Bu4^5^
134	Gamma-gliadin Bu-5^5^
323	Gamma-gliadin Bu-5^5^
335	Gamma-gliadin Bu-6^5^
527	Gamma-gliadin Bu-11^5^

385	Farinin Bu-1^2^
193	Farinin Bu-2^5^
207	Farinin Bu-2^2^
549	Farinin Bu-2^2^
336	Farinin Bu-3^5^
345	Farinin Bu-3^5^
542	Purinin Bu-1^5^
543	Purinin Bu-1^5^
219	Purinin Bu-2^2^
223	Purinin Bu-2^2^
220	Purinin Bu-3^2^
227	Purinin Bu-3^2^

99	Globulin-2 Bu-17366^2,4^
104	Globulin-2 Bu-17295^2^
106	Globulin-2 Bu-18428^2^
184	Globulin Glo-3 TC11_305389^3^
272	Globulin Glo-3 TC_234094^5^
14	HMW-GS Dx5 [GenBank:ABG68042]^2^
538	HMW-GS Dx5 [GenBank:ABG68042]^2^
25	HMW-GS Bx7 [GenBank:CAA32115]^2^
33	HMW-GS Bx7 [GenBank:CAA32115]^2^
514	HMWGS Dy10 [GenBank:P10387]^2^

94	Beta-amylase Bu-2^3^
93	Beta-amylase Bu-3^3^

232	Chitinase [GenBank:BAB18520]^2,4^
455	Chitinase [GenBank:AAX83262.1]^2,4^

248	Puroindoline-b [GenBank:AAT40244]^3^
271	Puroindoline-b [GenBank:AAT40244]^2^

89	ADP glucose pyrophosphorylase, large subunit [GenBank:CAD98749]^2^
295	Lipid transfer protein (LTP) Bu-2^2,4^
456	Thaumatin-like protein TC11_283136^2,4^
225	Triose phosphate isomerase [GenBank:CAC14917]^2,4^
311	Elongation factor EF1A [SwissProt:Q03033]^2,4^
202	Glucose and ribitol dehydrogenase RS_UWI_14903^2,4^
195	Gliadin, mixed spot^6^

^1 ^Identifications are from Dupont et al. [28].

^2 ^MS/MS analysis in Dupont et al. [28] indicated that spot contained only one protein.

^3 ^Predominant protein identified by MS/MS in Dupont et al. [28]. Analysis indicated that spot also contained other proteins from the same class.

^4 ^Predominant protein sequence identified in only one spot.

^5 ^Predominant protein identified by MS/MS in [28]. Analysis indicated that spot also contained proteins from a different class.

^6 ^More than one protein was identified in spot, but no one protein was predominant.

### Flour proteins that change in relative proportion with PAF

In the Dupont et al study [28], the same protein sequence was often associated with multiple 2-DE spots that were likely the result of charge trains, minor post-translational modifications or the lack of distinguishing peptides for the products of slightly different genes. Because it is important to consider the overall response of each protein, volumes of all spots associated with the same protein sequence were summed and compared under the two treatments (Additional file 2). The combined volumes of all spots associated with each of 54 unique protein sequences showed significant (p < 0.02) changes with PAF (Table 4).

**Table 4:** Unique proteins that changed significantly with PAF

			Spot Volume		
				
**Protein Sequence**^**1**^	# Spots	**Spot Numbers**^**2**^	-PAF	+PAF	% Change
Omega-gliadin *Gli-B3 *type^3^	6	60^+^, 63^+^, 69^+^,71^+^, 73^+^, 74^+^	1.940	5.070	161.4
Omega-gliadin *Gli-D3 *type^4^	2	476^+^, 477^+^	1.413	3.059	116.5
Omega-gliadin Bu-D5	2	135^+^, 391^+^	0.392	0.985	151.4
Omega-gliadin Cys type TC_262770	4	107^+^, 113^+^, 115^+^, 116	0.502	1.248	148.4

HMW-GS Ax2* [GenBank:AAB02788]	8	12^+^, 16, 17^+^, 18, 19^+^, 23, 24, 28	1.719	2.411	40.2
HMW-GS Bx7 [GenBank:CAA32115]	8	20^+^, 21^+^, 25^-^, 26, 27, 29, 33^-^, 302^+^	3.885	5.019	29.2
HMW-GS Dx5 [GenBank:ABG68042]	9	3^+^, 5, 7^+^, 10^+^, 14^-^, 36, 138, 307, 538^-^	2.691	3.732	38.7
HMW-GS 1By9 [GenBank:CAA43361]	8	40, 47, 52, 358, 349, 350^+^, 351, 360	1.478	2.277	54.0
HMWGS Dy10 [GenBank:P10387]	7	42^+^, 48, 51, 333, 407, 424, 514^-^	3.100	3.675	18.6

Alpha-gliadin Bu-1	1	342^+^	0.516	0.845	63.8
Alpha-gliadin Bu-3	1	468^+^	1.219	1.658	36.0
Alpha-gliadin Bu-4	1	467^+^	1.296	2.006	54.7
Alpha-gliadin Bu-12	5	328, 329, 387^+^, 524^+^, 525	1.903	3.063	60.9
Alpha-gliadin Bu-14	4	331, 190^+^, 206, 546	0.821	1.094	33.2
Alpha-gliadin Bu-BQ807130	1	124^+^	0.188	0.287	52.6

Serpin Bu-1	3	146, 147, 148^+^	0.461	0.599	30.1
Serpin Bu-1 or Bu-4	2	158, 162	0.069	0.040	-41.3
Serpin Bu-2	2	149^+^, 397^+^	0.067	0.193	188.6
Serpin Bu-3	2	398^+^, 399^+^	0.038	0.087	126.1
Serpin Bu-4	1	159^+^	0.146	0.236	62.2
Serpin Bu-5	2	151^+^, 154	0.181	0.268	48.5

Alpha-amylase inhibitor WMAI [PRF:223520]	2	289^-^, 528^-^	1.043	0.492	-52.9
Alpha-amylase inhibitor WDAI TC11_338524	1	283^-^	0.357	0.216	-39.6
Alpha-amylase inhibitor WDAI [GenBank:AAV91972]	1	286^-^	0.274	0.115	-57.9
Alpha-amylase inhibitor WDAI [SwissProt:P01085]	1	312^-^	1.305	0.671	-48.6
Alpha-amylase inhibitor WTAI-CM1 TC11_340510	1	313^-^	0.557	0.210	-62.3
Alpha-amylase inhibitor WTAI-CM2 [Swiss-Prot:P16851]	2	285^-^, 280^-^	1.377	0.479	-65.2
Alpha-amylase inhibitor WTAI-CM3 [Swiss-Prot:P17314]	2	264^-^, 265^-^	1.409	0.495	-64.9
Alpha-amylase inhibitor WTAI-CM16 [Swiss-Prot:P16159]	2	266^-^, 284^-^	1.235	0.512	-58.6
Alpha-amylase inhibitor WTAI-CM17 [Genbank:CAA42453]	2	274^-^, 282^-^	0.634	0.217	-65.8
Alpha-amylase/subtilisin inhibitor WASI [SwissProt:P16347]	1	244^-^	0.264	0.171	-35.0
Protease inhibitor CMx1/CMx3 TC11_309398	1	281^-^	0.361	0.229	-36.5
Protease inhibitor CMx1/CMx3 TC11_308146	1	290^-^	0.074	0.028	-62.8
Chymotrypsin inhibitor WCI [GenBank:CAD19440]	1	277^-^	0.327	0.116	-64.7

Farinin Bu-1	3	196, 385^-^, 386	0.358	0.246	-31.2
Farinin Bu-2	3	193^-^, 207^-^, 549^-^	1.271	0.325	-74.4
Farinin Bu-3	2	336^-^, 345^-^	0.815	0.336	-58.7

Purinin Bu-1	2	542^-^, 543^-^	0.487	0.294	-39.7
Purinin Bu-2	2	219^-^, 223^-^	0.524	0.261	-50.2
Purinin Bu-3	2	220^-^, 227^-^	0.491	0.270	-45.0

LMW-GS Bu-3	9	119^-^, 119a, 120, 131^+^, 132, 161^-^, 237^-^, 310, 316^-^	8.015	6.062	-24.4
LMW-GS Bu-6	1	173^-^	0.634	0.471	-25.7
LMW-GS Bu-7	3	144, 145^-^, 472	3.320	2.473	-25.5

Gamma-gliadin Bu-1	1	326^-^	0.191	0.132	-30.9

Globulin-2 Bu-18428	2	103, 106^-^	0.129	0.088	-31.6
Globulin-2 Bu-17366	1	99	0.043	0.028	-34.1

Chitinase [GenBank:BAB18520]	1	232^-^	0.095	0.041	-56.8
Chitinase [GenBank:AAX83262.1]	1	455^-^	0.125	0.047	-62.3

Puroindoline b [Genbank:AAT40244]	2	248^-^, 271^-^	0.512	0.251	-51.0
Beta-amylase Bu-2	2	94^-^, 462	0.231	0.145	-37.4
Lipid transfer protein (LTP) Bu-2	1	295^-^	0.117	0.035	-69.8
Thaumatin-like protein TC11_283136	1	456^-^	0.095	0.049	-48.4
Triose phosphate isomerase [GenBank:CAC14917]	1	225^-^	0.081	0.046	-42.6
Elongation factor EF1A [SwissProt:Q03033]	1	311^-^	0.168	0.113	-32.7
Glucose and ribitol dehydrogenase RS_UWI_14903	1	202^-^	0.117	0.004	-96.6

^1 ^Identifications are from Dupont et al. [28].

^2 ^Spots that increased or decreased significantly (p < 0.02) are indicated with + and -, respectively.

^3 ^Includes proteins identified as TC11_288652.

^4 ^Includes protein identified as [GenBank:AAT74547].

Proteins in four classes generally showed significant increases (Table 4). Some of the largest and most consistent changes were found among the omega-gliadins. Combined SVs for four different omega-gliadins ranged from 0.4 to 1.9 in the absence of PAF and 1.0 to 5.1 with PAF. Increases in SV ranged from 117% for the *Gli-D3 *omega-gliadins to 161% for the *Gli-B3 *omega-gliadins. The *Gli-B3 *omega-gliadins are of particular interest because they have been associated with the serious food allergy wheat-dependent exercise-induced anaphylaxis (WDEIA). Omega-gliadins that contain single cysteine residues and are likely incorporated into the glutenin polymer (omega-gliadin Cys type TC_262777) also increased in SV from 0.5 to 1.2, a 148% change. Combined spots associated with each of the five HMW-GS sequences also increased significantly in volume. SVs for individual HMW-GS types ranged from 1.5 to 3.9 in the absence of PAF and 2.3 to 5.0 with PAF (Table 4). Changes in SV ranged from a 19% increase for Dy10 to a 54% increase for By9. Only six alpha-gliadins increased significantly. Three alpha-gliadins, Bu-1, Bu-3 and Bu-4, contain multiple T-cell stimulatory epitopes involved in celiac disease and are encoded at the *Gli-D2 *locus [30]. SVs for these alpha-gliadins ranged from 0.5 to 1.3 in the absence of PAF and 0.8 to 2.0 with PAF, with increases of 36% to 64%. Bu-12 is an abundant alpha-gliadin encoded at the *Gli-B2 *locus that does not contain any of the major T-cell stimulatory epitopes or toxic sequences associated with celiac disease. The SV for this protein was 1.9 in the absence of PAF and 3.1 with PAF, a 61% increase. Alpha-gliadin Bu-14 is encoded at the *Gli-A2 *locus and contains the p31-43 sequence associated with the activation of the innate immune system in celiac disease [28]. This protein increased in total SV from 0.8 to 1.1, a 33% increase. Bu-BQ807130 is a minor alpha-gliadin that increased 53%. The serpins are the fourth group of proteins that generally showed increases in combined SVs. Spots associated with five serpin sequences showed significant increases in volume and ranged from 0.04 to 0.5 in the absence of PAF and 0.04 to 0.6 with PAF. Although serpin Bu-2 and Bu-3 showed 126 and 189% increases, respectively, both were relatively minor components of the flour. One minor serpin also showed a 41% decrease in total SV.

Proteins in three classes generally showed consistent decreases in SV (Table 4). Proteins associated with thirteen different sequences within the alpha-amylase/protease inhibitor family showed decreases in SV that ranged from 35 to 66%. SVs for these proteins ranged from 0.07 to 1.41 in the absence of PAF and 0.03 to 0.67 with PAF. The farinins and purinins also decreased consistently with PAF. SVs for the three farinins ranged from 0.36 to 1.27 in the absence of PAF and 0.25 to 0.34 with PAF while purinins ranged from 0.49 to 0.52 in the absence of PAF and 0.26 to 0.29 with PAF. Three LMW-GS also showed decreases of 24 to 26% with PAF. Bu-3, encoded at the *Glu-B3 *locus, is the most abundant protein in Butte 86 flour with SVs of 8.0 in the absence of PAF and 6.1 with PAF. Bu-7, encoded at the *Glu-D3 *locus, is also a major LMW-GS with SVs of 3.3 in the absence of PAF and 2.5 with PAF. LMW-GS Bu-6, encoded at the *Glu-A3 *locus, is a minor LMW-GS with SVs of only 0.6 in the absence of PAF and 0.5 with PAF. In contrast to most of the other gluten proteins, only one gamma-gliadin changed significantly with PAF. Bu-1 is a minor gamma-gliadin that decreased 31% in SV from 0.19 to 0.13. Two globulin-2 proteins that are relatively minor flour proteins also decreased significantly. Puroindoline b decreased 51% from 0.51 to 0.25 in total SV and beta-amylase Bu-2 decreased 37% from 0.23 to 0.15 in SV. Three defense proteins decreased, two chitinases by 57 and 62%, respectively, and LTP by 70%. Other proteins that decreased significantly included a thaumatin-like protein, triose phosphate isomerase, elongation factor EF1A and glucose and ribitol dehydrogenase. All had total SVs that were less than 0.2 in the absence of PAF and were found in single 2-DE spots.

### Overall effects of PAF on flour protein composition

Table 5 summarizes the effects of PAF on the accumulation of the different classes of wheat flour proteins. The omega-gliadins by far showed the greatest response to PAF with 13 of 15 spots and four of five protein sequences showing significant increases. Omega-gliadins encoded at the *Gli-A3f*, *Gli-B3h*, and *Gli-D3a *loci all showed significant changes (Additional file 3). Overall, the omega-gliadins increased 144% in total SV. HMW-GS increased 33% overall with significant changes in 16 of 40 spots and all five protein sequences. Alpha-gliadins also increased 31% overall even though only eight of 22 spots and six of 13 predominant sequences showed significant changes. Only alpha-gliadins encoded at the *Gli-B2 *and *Gli-D2 *loci showed significant changes (Additional file 3). Serpins increased 37% overall although significant changes were observed in only seven of 14 spots and five of seven protein sequences. The serpins are far less abundant than the glutenins and gliadins with combined SVs of only 1.2 in the absence of PAF and 1.6 with PAF. LMW-GS showed a 15% decrease in overall amount with 16 of 29 spots showing significant changes but only three of 11 protein sequences. When summed, only LMW-GS at the *Glu-B3h *and the *Glu-D3a *loci showed significant changes in SV with PAF (Additional file 3). Farinins, purinins, alpha-amylase/protease inhibitors and puroindolines all showed decreases overall that ranged from 45 to 63%. Beta-amylases also showed a 25% decrease even though significant changes were observed in only two of six spots and one of three protein sequences. Other enzymes also decreased by 29% even though only six of 23 spots and four of 20 protein sequences showed significant changes.

**Table 5:** Summary of changes in protein classes with PAF

				Total Spot Volume			**μg Protein/mg Flour**^**1**^		
					
	Total # Spots	**# Spots that Changed**^**2**^	**# Unique Proteins that Changed**^**2,3**^	-PAF	+PAF	**% Change**^**4**^	-PAF	+PAF	% Change
HMW-GS	40	16	5	12.87	17.11	32.9*	9.01	23.96	165.9
LMW-GS^5^	29	16	3	21.11	17.93	-15.1*	14.78	25.11	68.9
Alpha-gliadins^6^	22	8	6	15.58	20.41	31.0*	10.90	28.56	162.1
Gamma-gliadins^6^	16	7	1	12.82	12.10	-5.6	8.98	16.95	87.8
Omega-gliadins^6^	15	13	4	4.28	10.44	143.9*	3.00	14.62	387.8
Gliadin mixed spots^6,7^	5	2	n/a	2.12	2.56	20.3	1.49	3.58	140.7
Globulins	10	5	2	0.54	0.38	-30.4	0.38	0.53	39.2
Farinins	8	6	3	2.44	0.91	-62.9*	1.71	1.27	-25.7
Purinins	6	6	3	1.50	0.82	-45.1*	1.05	1.15	9.9
Triticins	7	1	0	1.35	1.50	10.9	0.95	2.10	121.9
GSPs and puroindolines	4	2	1	0.64	0.33	-49.0*	0.45	0.46	2.0
Serpins	14	7	5	1.15	1.58	37.4*	0.81	2.22	174.8
Amylase/protease inhibitors	19	19	13	9.55	4.09	-57.2*	6.69	5.73	-14.3
Other inhibitors	2	0	0	0.34	0.26	-24.2	0.24	0.37	51.6
Beta-amylase	6	2	1	0.66	0.50	-25.0*	0.46	0.70	49.9
Other enzymes	23	6	4	2.13	1.52	-28.8*	1.49	2.13	42.5
Other miscellaneous	5	3	3	0.55	0.34	-38.9	0.38	0.47	22.2
No ID	142	36	n/a	7.28	4.95	-58.3*	5.10	6.94	36.1

^1 ^Total protein was 70 μg/mg flour for -PAF and 140 μg/mg flour for +PAF; protein amount was calculated as (spot volume)*(total μg/mg flour)/100.

^2 ^Significant change, p < 0.02.

^3 ^n/a indicates not applicable.

^4 ^* indicates that overall change with PAF is significant (p < 0.02).

^5 ^LMW-GSs include only those with classic LMW-GS sequences.

^6 ^Gliadins include those with odd numbers of Cys that might be incorporated into the glutenin polymer.

^7 ^Spot contained a mixture of different proteins, but no one sequence was predominant.

The gluten proteins comprised a larger proportion of the total protein in flour produced with PAF. Combined SV for all gluten proteins was 68.8 in the absence of PAF and 80.6 with PAF. While the combined SV for the glutenins was similar under the two treatments, the combined SV for the gliadins increased from 32.7 to 43.0 with PAF. The ratio of HMW-GS to LMW-GS increased from 0.61 to 0.95. The ratio of gliadins to glutenins also increased from 1.02 to 1.30 with PAF.

### Relationship between response of proteins to PAF and S-amino acid content

**Figure 2:**
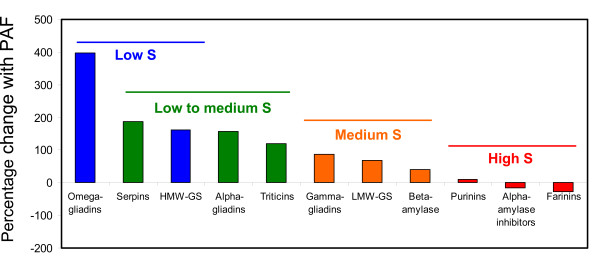
**Percentage change in absolute protein amount for protein types with different contents of Cys plus Met**. The content of S-containing amino acids was determined for each protein type (Additional file 4). Proteins containing low S are indicated in blue, low to medium S in green, medium S in orange and high S in red.

The analysis of spot volume data highlights the manner in which PAF alters the proportion of different proteins in the flour and is critical for understanding the effects of PAF on protein composition and flour quality. However, analysis of the data in terms of absolute protein amounts provides additional insight into effects of PAF on flour composition. It is important to recognize that total protein content doubled from 70 to 140 μg/mg flour with PAF. Thus, even those proteins that showed no change in SV doubled in amount per unit of flour. Such changes in amount as well as proportion may have significant effects on flour quality. Changes in absolute amounts of each protein type also provide insights into the responses of genes within the different classes. When the spot volume data was converted to absolute protein amounts, it is clear that there was an increase in the absolute amount of protein in most classes (Table 5). Exceptions were the farinins that decreased 26% in absolute amount, the alpha-amylase/protease inhibitors that decreased 14% and the puroindolines that showed relatively little change. The degree of change in the different protein classes seemed to be related to the proportions of S-containing amino acids Cys and Met in the mature proteins from each class (Figure 2). The content of the S-containing amino acids ranged from 0% for most omega-gliadins to 12.3% for an alpha-amylase inhibitor sequence (Additional file 4). The biggest increase with PAF was for the low S omega-gliadins (0% to 0.6% Cys plus Met). There were moderate increases for the low-to-medium S serpins (3.2% Cys plus Met), the low S HMW-GSs (0.9% to 1.7% Cys plus Met), the low-to-medium S alpha-gliadins (2.4% to 3.1% Cys plus Met), and triticins (2.5% Cys plus Met). There were lesser increases for gamma-gliadins (3.6% to 5.3% Cys plus Met), LMW-GS (3.7% to 5.1% Cys plus Met), and beta-amylase (3.5 to 4.1% Cys plus Met). The high S purinins (6.5% to 6.6% Cys plus Met) showed relatively little change, high S alpha-amylase/protease inhibitors (9.2% to 12.3% Cys plus Met) decreased slightly and high S farinins (9.8 to 11.2% Cys plus Met) decreased. There was not a clear relationship between absolute protein amount and Gln plus Pro or Lys content, although Gln plus Pro was highest for those proteins with the lowest amount of Cys plus Met.

## Discussion

A 2-DE approach was used to quantify the proportions of individual proteins in flour from grain produced with and without PAF. The study defines the effects of PAF more precisely than prior studies in terms of specific proteins and protein classes and forms a baseline for other 2-DE studies that explore the effects of environmental variables on flour protein composition. The application of PAF significantly increased flour protein content and substantially altered flour protein composition, both of which can impact flour functional properties and quality. More than one third of the total number of individual proteins identified in wheat flour by Dupont et al [28] showed significant changes in SV with PAF. There was an increase in the proportion of HMW-GS and decrease in the proportion of LMW-GS in the flour with PAF and an increase in ratio of HMW-GS to LMW-GS. The proportion of the two types of glutenin subunits in flour is thought to be an important indicator of flour quality because it can affect the nature of large glutenin polymers that confer elasticity to dough made from wheat flour. The ratio of gliadins to glutenins also increased with PAF, due in part to increased proportions of omega- and alpha-gliadins. The gliadins are thought to confer extensibility to dough made from wheat flour, but the roles of specific types of gliadins in flour quality have not been well defined. The data further demonstrated that alpha-gliadins from the *Gli-D2 *locus containing multiple celiac epitopes and those from the *Gli-B2 *locus that do not contain celiac epitopes increased to similar degrees. Certain alpha-, gamma- and omega-gliadins from Butte 86 also contain odd numbers of Cys residues and are likely to be incorporated into the glutenin polymer [29,30,32]. Of those gliadins with an odd number of Cys, only the omega-gliadins changed in response to PAF.

The work demonstrates that it is possible to use one flour extract to distinguish among highly similar gluten proteins while also quantifying minor flour protein components such as serpins, triticins, farinins, purinins, alpha-amylase/protease inhibitors, globulins, puroindolines and enzymes. Serpins increased in proportion with PAF while the farinins, purinins, puroindolines and amylase/protease inhibitors decreased. There was little change in the proportions of triticins and globulins and only a handful of enzymes decreased with PAF. These included two enzymes involved in glucose degradation (beta-amylase and glucose and ribitol dehydrogenase), one involved in glycolysis (triose phosphate isomerase) and one involved in defense (chitinase). Our results are more detailed and precise but are in general agreement with previous studies that distinguished among protein types based on protein fractionation, banding patterns in SDS-PAGE and retention times in RP-HPLC. Most studies separated glutenin, gliadin and albumin/globulin or amphiphilic fractions before quantifying them, as it has been difficult to distinguish among these proteins in total flour extracts. However, protein fractionation introduces a trade-off between the purity of each fraction and the amount of protein recovered. Also, a single protein may partition into more than one fraction, and environmental treatments may alter partitioning of proteins among fractions. In contrast, the present study measured the effects of PAF on individual, genetically identifiable proteins without subjecting them to fractionation and thereby provides a valuable overview of the effect of PAF on the major components of the flour.

It is notable that a number of the proteins that changed in proportion with PAF are likely food allergens. Proteins designated as food allergens in the Allergome database [33] that increased in proportion with PAF include the *Gli-B3 *omega-gliadins (also referred to as the omega-5 gliadins) and the serpins while those that decreased include the defense proteins LTP, chitinase, the alpha-amylase inhibitor WTAI-CM3, and thaumatin-like protein. Globulin-2 also has substantial similarity to known food allergens in other plants [34]. Thus, the application of PAF altered the allergenic potential of the flour. In a previous study of developing endosperm from Butte 86, some of the same proteins also changed in proportion in response to high temperatures when plants were supplied with PAF [35]. Interestingly, LTP, chitinase and globulin-2 increased in proportion with high temperature while the proportions of several serpins decreased. These studies illustrate the complex regulation of genes for different wheat flour proteins and highlight the importance of considering effects of the fertilizer regimen in all experiments.

When considered in terms of absolute protein amounts, it is clear that the μg protein per mg flour increased for all gluten proteins as well as for many of the other protein classes. The gliadins and glutenins have a high content of Gln and Pro and serve as efficient reservoirs for storing seed N. As the N-supply increased, the synthesis of gliadins and glutenins increased, particularly ones that were low in S. This is consistent with the observed increase in the ratio of N to S in the flour. The response of the farinins to PAF was very different from that of the major gluten proteins. The farinins have moderately high levels of Gln and Pro (31-35%) and structural similarities to the major wheat gluten proteins. Yet their absolute amounts did not increase with PAF. Instead, their response to PAF was similar to that of the high S alpha-amylase/protease inhibitors.

The mechanisms by which wheat flour proteins respond to PAF are undoubtedly complex. Overall, it appeared that the response to PAF was related to the S content of the proteins with low S proteins showing the greatest increase with PAF. However, individual proteins within each class were accumulated to different levels, suggesting different basal levels of expression of the corresponding genes. Additionally, there was a differential response to PAF among individual proteins within certain classes, most notably the alpha-gliadins and LMW-GS. For the omega-gliadins, changes in protein levels are in accord with the results of transcript studies in Butte 86 [16,32]. In previous studies, PAF did not affect the temporal pattern of transcript accumulation but resulted in increased transcript levels for omega-gliadins detected by hybridization analysis and by quantitative reverse transcription polymerase chain reaction (qRT-PCR) along with increased rates of omega-gliadin protein accumulation [15,16]. This is in agreement with the large increases in omega-gliadin protein reported here. The same qRT-PCR study showed that PAF had little effect on transcript levels of a gamma-gliadin gene [32]. Thus far, transcript levels of HMW-GS, LMW-GS and alpha-gliadins in Butte 86 have been assessed only by hybridization analysis [16]. Further studies are needed to examine the effects of PAF on the expression of individual gluten protein genes during wheat grain development and to address the relationships between promoter structures for these genes, differences in basal levels of gene expression and the differing effects of N on protein accumulation.

While 2-DE is a powerful method for evaluating changes in flour protein profiles in response to specific treatments, the data is complex and needs to be considered on several levels. Frequently, 2-DE studies report changes in individual spots that are subsequently identified by MS/MS. This approach reveals only part of the overall picture. Recent studies indicate that the same protein sequence can be distributed among several different 2-DE spots [28,36-39] and that all spots associated with a unique protein sequence may not show the same response. While it is not known whether the origin of these multiple spots is biologically significant or due to extraction and/or analytical techniques [28], it nonetheless is important to consider each change in the context of the proteome being analyzed. In this study, individual spots associated with 77 protein sequences showed significant changes in SV with PAF. However, when the volumes for all spots containing the same unique proteins were summed, changes in only 54 distinct protein sequences were significant. Additionally, when MS/MS methods are optimized to generate large numbers of peptides for each spot as was done in the Dupont et al. [28] study, it is apparent that single 2-DE spots often contain more than one protein sequence. In most cases, the majority of peptides obtained by MS/MS from a spot could be assigned to a single sequence that was designated as the predominant protein in the spot. For example, 45 peptides were identified by MS/MS for spot 524 [28]. Thirty-two were assigned to alpha-gliadin Bu-12 resulting in 80% MS/MS coverage, while the remaining nine peptides were distributed among three other alpha-gliadin sequences. It is certainly possible that the changes observed in this spot with PAF are due to changes in the levels of minor proteins in the spot rather than to the predominant protein. MS/MS analysis of equivalent spots from both treatments could help to resolve this issue. Finally, it is not always possible to identify all of the protein spots that respond to a treatment. In this study, we were unable to obtain valid identifications for 36 spots that changed significantly with PAF, all of which represented minor components of the flour. The failure to identify these proteins may be because they were of very low abundance. It is also possible that the proteins did not cleave well with the proteases used for MS/MS identification, the peptides were not detected by the mass spectrometer or the corresponding protein sequences were not present in the database searched with MS/MS data. Further studies of fractionated flour proteins may complement this study and identify additional minor components that may be of interest. Despite limitations of the 2-DE approach, this study provides new insight into the effects of PAF on individual flour proteins that are important for flour quality and allergenic potential.

## Conclusions

Millers and bakers encourage scientists to produce cultivars with increased stability of flour quality across years and growth environments. However, to select targets for breeding programs or biotechnology efforts it is important to first identify the genes and proteins that contribute to variability in quality. The current study used quantitative 2-DE to address the effects of PAF on the entire array of abundant flour proteins. The results indicate that omega-gliadins are very sensitive to PAF and could be a major source of environmental variability. HMW-GSs, serpins, certain alpha-gliadins and LMW-GSs also appear to contribute to this variability. The study is essential for predicting the effects of agronomic inputs on flour composition and establishes the foundation for future work aimed at deciphering the complex effects of temperature and drought on the wheat flour proteome.

## Methods

### Plant materials

Plants of the US hard red spring wheat *Triticum aestivum *'Butte 86' were grown in a climate-controlled greenhouse with 16 hr days and 8 hr nights at 24°C maximum daytime and 17°C minimum night-time temperatures [1,28]. Prior to anthesis, plants were watered by drip irrigation with 0.6 g.l^-1 ^Plantex fertilizer (NPK, 20:20:20), at approximately 0.1 g each of N, P and K per pot per day. Plants were grown in 36 pots of 25 cm diameter at a density of seven plants per pot. At anthesis, pots were divided into six groups. Three sets of pots were flushed with water to remove remaining NPK and subsequently were hand-watered without fertilizer. The other three sets of pots received drip irrigation with NPK fertilization, at approximately 0.3 g each of N, P and K per pot per day. Sets with and without fertilizer were alternated spatially in the greenhouse, and pots within a set were rotated weekly. Pots were weighed daily and water added to maintain the pots at 80% of water capacity. Grain was harvested at maturity and samples were milled to white flour with a Brabender Quadrumat Junior (South Hackensack, NJ) at the Hard Winter Wheat Quality Laboratory (US Department of Agriculture, Agricultural Research Service, Manhattan, KS) and tested for mixing and baking quality as previously described [15]. Subsequently, flour was stored at -80°C. Flour N, C and S were determined by combustion analysis of 35 mg samples, in triplicate, using an Elementar Vario Macro Elemental Analyzer (Hanau, Germany) in CNS mode with a sulfadiazine standard. Protein was calculated as 5.7 times N.

### 2-DE of total flour proteins

Three separate samples of flour from each biological replicate were extracted and analyzed by 2-DE (18 gels total) as described in detail previously [28,40,41]. Briefly, total flour protein was extracted with SDS under reducing conditions, precipitated with acetone and protein amount determined by the method of Lowry et al. [42]. Dried protein was solubilized in urea buffer consisting of 9 M urea, 4% NP-40, 1% DTT and 2% ampholytes at a concentration of 3 μg/μl, then 18 μg was loaded onto each IEF gel, focused, and IEF gels were frozen at -70°C. Proteins were separated in the second dimension by SDS gel electrophoresis using Novex NuPage 4-12% acrylamide Bis-Tris gels with MES/SDS running buffer (Invitrogen Corporation, Carlsbad, CA). Gels were stained with Coomasie G-250 (Sigma, St. Louis, MO), destained in water for 2 h and stored at 4°C in 20% ammonium sulfate. Gels were scanned and spots were matched between gels, quantified and normalized using computer software (Progenesis PG240 v 2006, Nonlinear Dynamics, Newcastle upon Tyne, UK) as described in detail [28]. All proteins were identified previously by MS/MS [28]. Spot volumes of identified protein spots were also reported in [28]. Visual inspection of identified spots matched between gels by computer software detected no matching errors.

### Statistical analysis

The experimental plan included two treatments and three biological replicas, obtained from the three sets of six individual pots for each treatment. For each of the resulting six flour samples, 2-DE was performed in triplicate. The 2-DE analysis software computed the SV for each spot as the average of the three replicate gels. These average SVs for the three biological replicates and two treatments were then subjected to analysis of variance for each spot with SAS software (SAS Institute Inc. 2009. SAS OnlineDoc^® ^9.2. Cary, NC: SAS Institute Inc.). Means and standard deviations for all proteins were similar among biological replicates. Probabilities from an F-test comparing means of the two treatments were calculated, both assuming that the variances were similar enough to be pooled for both treatments (4 error degrees of freedom) or differed enough between the treatments that pooling would not be appropriate (2 error degrees of freedom). An F-test was used comparing the two error terms to determine which mean comparison test was more appropriate. A Bonferroni adjustment to the F probability was also made to account for the increased likelihood of chance significance in a large set of tests. Similar calculations were made for the sums of the SVs for specific protein sequences and specific protein types, in which the average SVs for each spot for a biological replica were summed and then these sums for the three biological replicas were used in computing the analysis of variance for each protein sequence or protein type. Appropriate calculations are shown in Additional files 1, 2 and 3; generally the calculation using the simplest assumption was used for tables in the main document.

The absolute amount of each protein per mg of flour was estimated by multiplying SV by total protein per mg of flour. Proportions of Cys, Met, Lys, Pro and Gln were calculated as the percentage of total amino acids in the mature proteins using published sequences from Butte 86 [28-30] or sequences from NCBI. Because most omega-gliadin sequences are incomplete, this information was determined from amino acid analysis of purified proteins [43].

## Abbreviations

2-DE: 2-dimensional gel electrophoresis; HMW-GS: high molecular weight glutenin subunit; LMW-GS: low molecular weight glutenin subunit; MS/MS: tandem mass spectrometry; N: nitrogen; NPK: nitrogen, phosphorus, potassium 1:1:1 fertilizer; PAF: post-anthesis fertilization; RP-HPLC: reverse phase-high pressure liquid chromatography; S: sulfur; SDS-PAGE: SDS polyacrylamide gel electrophoresis; SV: normalized spot volume.

## Competing interests

The authors declare that they have no competing interests.

## Authors' contributions

FD and SA designed the study, analyzed and interpreted the data and drafted the manuscript; CT carried out the quantitative 2-DE gel analysis; WH participated in the 2-DE gel analysis; LW advised on experimental design and performed statistical analysis and interpretation; WV provided the original MS/MS analysis that was essential to the project. All authors read and approved the manuscript.

## Supplementary Material

Additional file 1 **Statistical analysis for all individual 2-DE spots**. Click here for file

Additional file 2 **Statistical analysis for summed spot volumes for each unique protein sequence**. Click here for file

Additional file 3 **Statistical analysis for summed spot volumes for each protein class**. Click here for file

Additional file 4 **Proportions of key amino acids in flour protein types based on the sequences of the mature proteins**. Click here for file
