# Supplementary material for: Differential effects of a post-anthesis fertilizer regimen on the wheat flour proteome determined by quantitative 2-DE
Source: Proteome Sci. 2011 Aug 4;9:46. doi: 10.1186/1477-5956-9-46 (PMC3168407; doi:10.1186/1477-5956-9-46)
Supplement: Additional file 4 — Proportions of key amino acids in flour protein types based on the sequences of the mature proteins. [file 1477-5956-9-46-S4.DOCX]

Additional file 4. Proportions of key amino acids in flour protein types based on the sequences of the mature proteins

| Protein type | Cys + Met | Gln + Pro | Lys |
| --- | --- | --- | --- |
|  | (%) | (%) | (%) |
| Low S |  |  |  |
| Omega-gliadins^1^ | 0.0 to 0.6 | 68.5 to 73.1 | 0.3 to 0.5 |
| HMW-GS | 0.9 to 1.7 | 43 to 47 | 0.7 to 1.1 |
| Low-to-medium S |  |  |  |
| Alpha-gliadins | 2.4 to 3.1 | 49 to 53 | 0 to 0.7 |
| Triticins | 2.5 | 17.3 | 3.6 |
| Serpins | 3.2 | 9.7 | 4.8 |
| Medium S |  |  |  |
| Beta-amylases | 3.5 to 4.1 | 11 to 12 | 3.3 to 4.0 |
| Gamma-gliadins | 3.6 to 5.3 | 49 to 56 | 0.4 to 1.1 |
| LMW-GS | 3.7 to 5.1 | 43 to 54 | 0.3 to 1.4 |
| High S |  |  |  |
| Purinins | 6.5 to 6.6 | 29 to 31 | 1.6 to 2.2 |
| Farinins | 9.8 to 11.2 | 31 to 35 | 0.7 to 2.3 |
| Alpha-amylase inhibitor^2^ | 9.2 to 12.3 | 9.2 to 16.1 | 1.7 to 4.9 |

^1^Determined from amino acid analysis of purified omega gliadins [43].

^2^ Alpha-amylase inhibitors WCI [Genbank:CAD19440], WDAI [Genbank:AAV91972], WDAI [Swiss-Prot:P01085], WMAI [PRF:223520]; WTAI-CM2 [Swiss-Prot:P16851], WTAI-CM3 [Swiss-Prot:P17314], WTAI-CM16 [Swiss-Prot:P16159], WTAI-CM17 [Genbank:CAA42453].
